# Overexpression of CIP2A is an independent prognostic indicator in nasopharyngeal carcinoma and its depletion suppresses cell proliferation and tumor growth

**DOI:** 10.1186/1476-4598-13-111

**Published:** 2014-05-19

**Authors:** Na Liu, Qing-Mei He, Jie-Wei Chen, Ying-Qin Li, Ya-Fei Xu, Xian-Yue Ren, Ying Sun, Hai-Qiang Mai, Jian-Yong Shao, Wei-Hua Jia, Tie-Bang Kang, Mu-Sheng Zeng, Jun Ma

**Affiliations:** 1 Sun Yat-sen University Cancer Center; State Key Laboratory of Oncology in South China; Collaborative Innovation Center of Cancer Medicine, 651 Dongfeng Road East, Guangzhou, People’s Republic of China

**Keywords:** Nasopharyngeal carcinoma, CIP2A, Proliferation, Cell growth, Survival

## Abstract

**Background:**

Cancerous inhibitor of protein phosphatase 2A (CIP2A) is an oncoprotein that acts as a prognostic marker for several human malignancies. In this study, we investigated the clinical significance of CIP2A and its function in nasopharyngeal carcinoma (NPC).

**Methods:**

Quantitative RT-PCR, western blot, and immunohistochemistry analyses were used to quantify CIP2A expression in NPC cell lines and clinical samples. Kaplan-Meier curves were used to estimate the association between CIP2A expression and patient survival. The functional role of CIP2A in NPC cell lines was evaluated by small interfering RNA-mediated depletion of the protein followed by analyses of cell proliferation and xenograft growth.

**Results:**

CIP2A levels were upregulated in NPC cell lines and clinical samples at both the mRNA and protein levels (*P* < 0.01). Patients with high CIP2A expression had poorer overall survival (HR, 1.98; 95% CI, 1.16-3.34; *P* = 0.01) and poorer disease-free survival (HR, 1.68; 95% CI, 1.07-2.62; *P* = 0.02) rates than patients with low CIP2A expression. In addition, CIP2A expression status was an independent prognostic indicator for NPC patients. The depletion of CIP2A expression inhibited c-Myc protein expression in NPC cell lines, suppressed cell viability, colony formation, and anchorage-independent growth in vitro, and inhibited xenograft tumor growth in vivo.

**Conclusions:**

Our data demonstrate that high CIP2A expression in patients was associated with poor survival in NPC, and depletion of CIP2A expression inhibited NPC cell proliferation and tumor growth. Thus, these results warrant further investigation of CIP2A as a novel therapeutic target for the treatment of NPC.

## Introduction

Nasopharyngeal carcinoma (NPC) is an epithelial malignancy of the nasopharynx, and global statistics obtained for different world regions reveal that its distribution is extremely unbalanced, with the highest incidence rates occurring in Southern China [1,2]. According to data from the International Agency for Research on Cancer, there were an estimated 84,000 cases of NPC and 51,600 NPC-related deaths in 2008 [3]. Although the increased prevalence of intensity-modulated radiation therapy with concurrent chemoradiation therapy has improved the local and regional control of NPC for patients with locoregionally advanced disease, their prognosis is still poor due to recurrence and/or distant metastasis [4,5]. Thus, a better understanding of the underlying molecular mechanisms involved in NPC pathogenesis and progression is essential for the development of novel therapeutic strategies to treat patients with NPC.

Cancerous inhibitor of protein phosphatase 2A (CIP2A), also known as KIAA1524 and p90, is a recently identified human oncoprotein that inhibits the degradation of c-MYC by inhibiting the protein phosphatase 2A (PP2A)-mediated dephosphorylation of MYC at serine 62 [6-8]. In addition, CIP2A and MYC appear to be regulated by a positive feedback loop that promotes the expression of both proteins [7]. Originally identified as an oncoprotein, CIP2A contributes to the immortalization and malignant transformation of human cells [6], and the depletion of CIP2A decreased cell viability and anchorage-independent growth in a variety of human malignancies [6-11]. More importantly, CIP2A was recently found to be overexpressed at a high frequency in most types of cancer and may serve as a prognostic predictor [12-20]. However, the clinical significance and biological function of CIP2A in NPC has not been thoroughly investigated to date.

In the present study, we examined both the mRNA and protein expression levels of CIP2A in NPC cell lines and tissue samples and further analyzed the clinical significance of CIP2A in a cohort of NPC patients. In addition, we explored the potential role of CIP2A in NPC cell proliferation and tumor growth, which could help to better understand the pathology of NPC and may further provide a novel therapeutic target for the treatment of NPC patients.

## Results

### Expression of CIP2A in NPC cells and tissues

**Figure 1:**
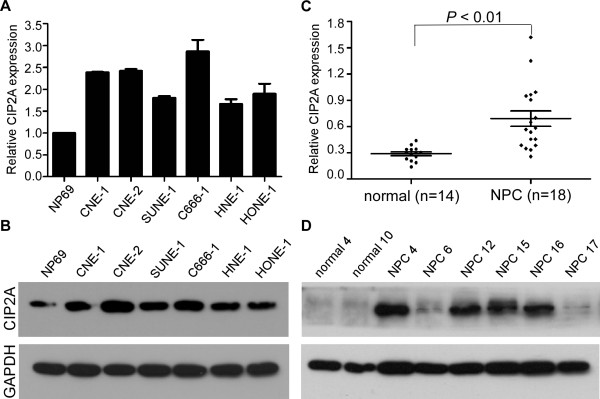
**Expression levels of CIP2A in NPC cell lines and clinical samples. (A-B)** Expression levels of CIP2A mRNA **(A)** and protein **(B)** in NP69 and NPC cell lines. **(C-D)** Expression levels of CIP2A mRNA **(C)** and protein **(D)** in NPC tissues and normal nasopharyngeal epithelial tissues. GAPDH was used as the endogenous control. Data are presented as the mean ± SD, and *P* values were calculated with Student’s *t*-test.

Quantitative RT-PCR and western blot analyses were used to determine the levels of CIP2A mRNA and protein in NPC cell lines and the normal nasopharyngeal epithelial cell line NP69. CIP2A was significantly upregulated in all six NPC cell lines when compared to the NP69 cells at both the mRNA and protein levels (Figure 1A-B). Furthermore, we detected CIP2A mRNA expression in 18 freshly frozen NPC tissues and 14 normal nasopharyngeal epithelial tissues and found that CIP2A mRNA levels were considerably higher in NPC tissues (Figure 1C). Similarly, CIP2A protein was also increased in NPC tissues when compared to normal nasopharyngeal epithelial tissues (Figure 1D). These results suggest that CIP2A is upregulated in NPC.

### CIP2A expression and the clinical variables of NPC patients

**Figure 2:**
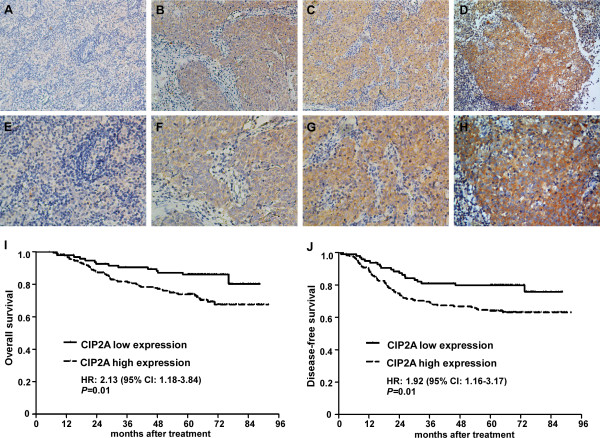
**Expression levels of CIP2A and survival of NPC patients. (A-H)** Dicer1 protein expression is mainly localized to the cytoplasm. **(A, E)** Negative staining (**A**: 200×; **E**: 400×); **(B, F)** Weak staining: light yellow (**B**: 200×; **F**: 400×); **(C, G)** Moderate staining: yellow brown (**C**: 200×; **G**: 400×). **(D, H)** Strong staining: brown (**D**: 200×; **H**: 400×). **(I-J)** Patients with high CIP2A expression had poorer overall survival **(I)** and poorer disease-free survival **(J)** rates than patients with low CIP2A expression.

We then analyzed CIP2A protein expression levels in a set of 280 paraffin-embedded NPC tissue samples using immunohistochemistry. Representative staining of CIP2A in NPC tissue is shown in Figure 2A-H, and positive staining of CIP2A was mainly observed in the cytoplasm. The presence of CIP2A protein was detected in 254 of the 280 (90.3%) cancer samples analyzed, and CIP2A protein expression was highly expressed in 184 of the 280 (65.7%) NPC patients examined. Furthermore, patients with high CIP2A expression exhibited a significant association with T stage (*P* = 0.04), TNM stage (*P* < 0.01), distant metastasis (*P* < 0.01), and patient death (*P* = 0.01). There were no significant associations between CIP2A expression and patient age, sex, WHO type, VCA-IgA, EA-IgA, N stage, or locoregional failure (Table 1).

**Table 1:** Clinical characteristics of NPC patients according to high and low CIP2A expression

**Characteristics**	**No. of patients**	**Expression of CIP2A**		***P*** **value**
		**Low, n (%)**	**High, n (%)**	
**Age**				
≤45	130	48 (50)	82 (45)	0.39
>45	150	48 (50)	102 (55)	
**Sex**				
Male	208	72 (75)	136 (74)	0.84
Female	72	24 (25)	48 (26)	
**WHO type**				
I + II	10	2 (2)	8 (6)	0.50
III	270	94 (98)	176 (94)	
**VCA-IgA**				
< 1:80	41	16 (17)	25 (14)	0.49
≥ 1:80	239	80 (83)	159 (86)	
**EA-IgA**				
< 1:10	68	24 (25)	44 (24)	0.84
≥ 1:10	212	72 (75)	140 (76)	
**T Stage**				
T1-T2	137	55 (57)	82 (45)	0.04
T3-T4	143	41 (43)	102 (55)	
**N Stage**				
N0-N1	173	61 (64)	112 (61)	0.66
N2-N3	107	35 (36)	72 (39)	
**TNM Stage**				
I-II	86	40 (42)	46 (25)	<0.01
III-IV	194	56 (58)	138 (75)	
**Locoregional failure**				
Yes	38	11 (11)	27 (15)	0.46
No	242	85 (89)	157 (85)	
**Distant metastasis**				
Yes	63	11 (11)	52 (28)	<0.01
No	217	85 (89)	132 (72)	
**Death**				
Yes	67	14 (15)	53 (29)	0.01
No	213	82 (85)	131 (71)	

### CIP2A expression and survival of NPC patients

Kaplan-Meier analysis and the log-rank test were used to calculate the effects of CIP2A on survival, and the results indicated that patients with high CIP2A expression were significantly associated with poorer overall and disease-free survival rates than patients with low CIP2A expression (all *P* < 0.01, Figure 2I-J). The cumulative 5-year survival rate was 86.5% (95% CI, 79.7-93.3) in the low-CIP2A-expression group, whereas it was only 74.5% (95% CI, 68.2-80.8) in the high-CIP2A-expression group. CIP2A expression, TNM stage, sex, age, WHO type, and EBV seromarkers were analyzed using univariate and multivariate Cox regression analyses. Univariate analyses indicated that patients with high CIP2A expression and advanced disease stages exhibited worse outcomes than those with low CIP2A expression (Table 2). Multivariate analyses revealed that CIP2A expression and TNM stage were independent prognostic indicators in NPC patients (all *P* < 0.05, Table 2).

**Table 2:** Univariate and multivariable Cox regression analyses of CIP2A expression levels and survival rates

	**Univariate analysis**			**Multivariate analysis**		
**Variable**	**HR**	**95% CI**	***P-*** **value**	**HR**	**95% CI**	***P-*** **value**
		**Overall survival**				
CIP2A expression (High vs. Low)	2.13	1.18-3.84	0.012	1.85	1.02-3.35	0.042
TNM stage (III-IV vs. I-II)	3.16	1.57-6.39	0.001	2.87	1.42-5.82	0.003
Sex (Male vs. Female)	1.57	0.86-2.87	0.146			
Age (>45 years vs. ≤45 years)	1.43	0.87-2.34	0.155			
WHO type (III vs. I + II)	0.81	0.26-2.58	0.722			
VCA-IgA (≥1:80 vs. < 1:80)	1.80	0.78-4.17	0.168			
EA-IgA (≥1:10 vs. < 1:10)	1.19	0.66-2.15	0.555			
		**Disease-free survival**				
CIP2A expression (High vs. Low)	1.92	1.16-3.17	0.011	1.70	1.03-2.82	0.039
TNM stage (III-IV vs. I-II)	2.87	1.59-5.17	<0.001	2.59	1.43-4.69	0.002
Sex (Male vs. Female)	1.57	0.93-2.68	0.094			
Age (>45 years vs. ≤45 years)	1.25	0.82-1.93	0.301			
WHO type (III vs. I + II)	0.78	0.29-2.12	0.622			
VCA-IgA (≥1:80 vs. < 1:80)	2.06	0.95-4.46	0.068			
EA-IgA (≥1:10 vs. < 1:10)	1.44	0.84-2.48	0.190			

### Effects of CIP2A depletion on MYC expression and cell proliferation

**Figure 3:**
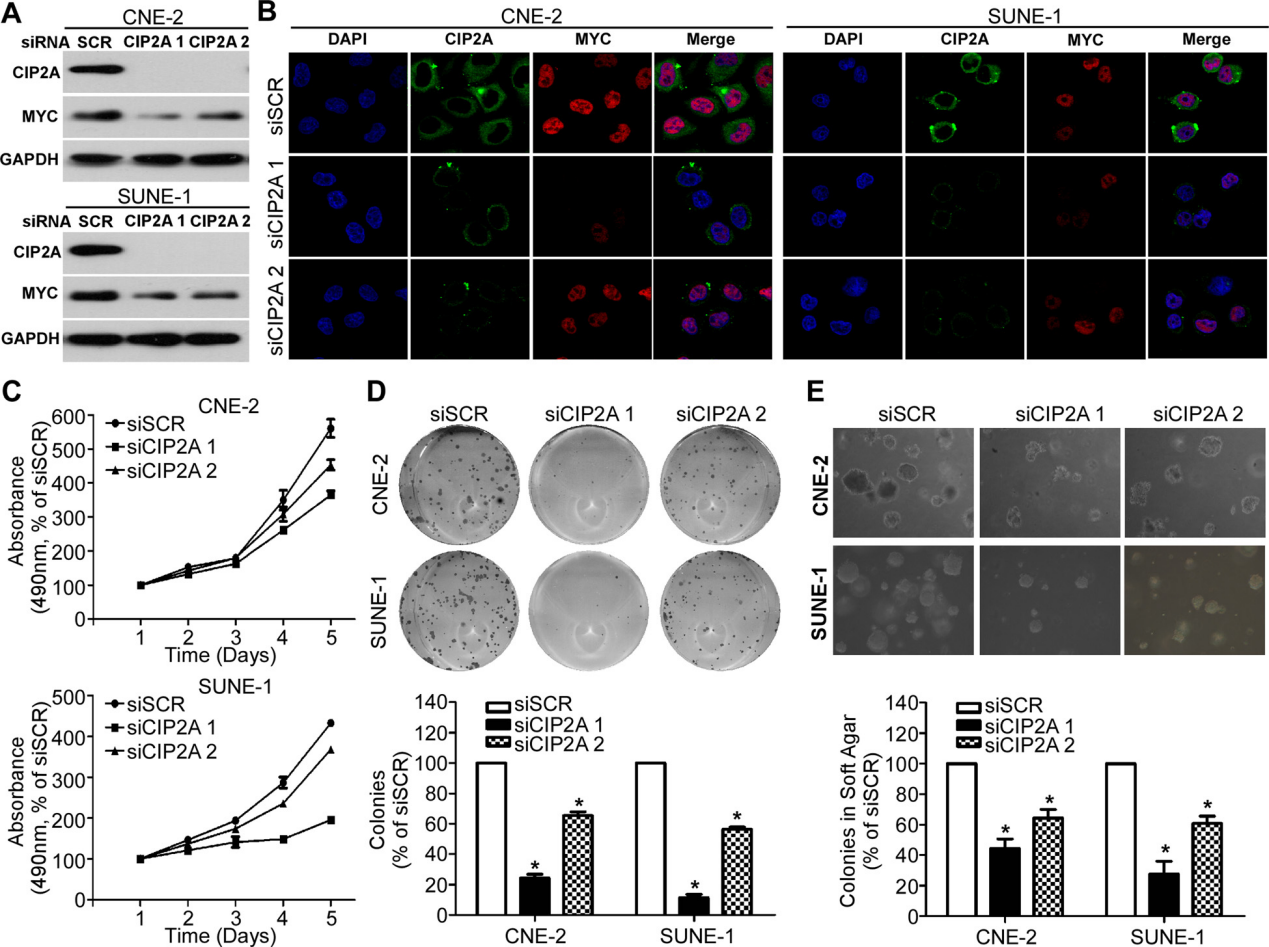
**Effects of CIP2A depletion on MYC expression and NPC cell proliferation in vitro. (A-B)** Effects of CIP2A siRNA on CIP2A and MYC protein expression in CNE-2 and SUNE-1 cells detected by western blot analysis **(A)** and immunofluorescence staining **(B)**; **(C-E)** Effects of CIP2A siRNA on the cell viability **(C)**, proliferation **(D)**, and anchorage-independent growth **(E)** of CNE-2 and SUNE-1 cells. Data are presented as the mean ± SD. *P < 0.05 compared to the control using Student’s *t*-test.

CIP2A protein expression was remarkably inhibited in CNE-2 and SUNE-1 cells treated with siRNA specifically directed against CIP2A when compared to those treated with scrambled control siRNA (Figure 3A-B). More importantly, depletion of CIP2A by siRNA suppressed the MYC protein expression in both CNE-2 and SUNE-1 cells (Figure 3A-B). We also studied the effects of CIP2A depletion on cell viability and proliferation ability using MTT assays and colony formation assays. CNE-2 and SUNE-1 cells transfected with siCIP2A displayed significant growth inhibition compared to those transfected with scrambled control siRNA (*P* < 0.05, Figure 3C). Moreover, cells transfected with siCIP2A exhibited fewer and smaller colonies compared to the controls (*P* < 0.05, Figure 3D).

### Effects of CIP2A depletion on tumor growth

**Figure 4:**
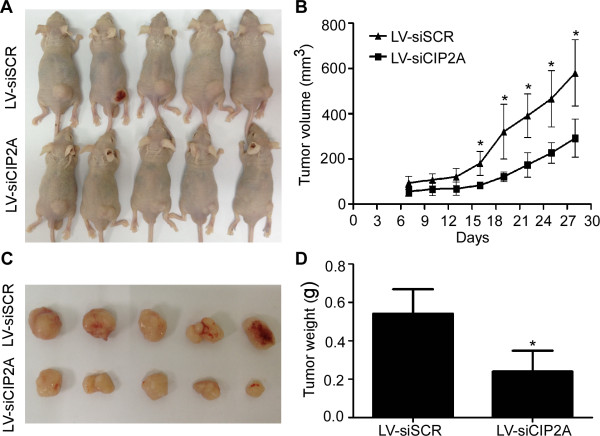
**Effects of CIP2A depletion on NPC xenograft tumor growth in vivo. (A)** SUNE-1 cells stably expressing shCIP2A or scrambled control siRNA were subcutaneously injected into nude mice. SUNE-1 cells stably expressing shCIP2A had smaller tumors than scrambled controls after four weeks. **(B)** The growth curves of tumor volumes. **(C)** Representative picture of xenograft tumors. **(D)** Tumor weight. Data are presented as the mean ± SD. *P < 0.05 compared to the control using Student’s *t*-test.

The ability of cells to grow and form colonies on soft agar is a hallmark characteristic of malignantly transformed cells. To study the effects of CIP2A on the malignant growth of NPC cells, we transfected siCIP2A or scrambled control siRNA into CNE-2 and SUNE-1 cells and found that CIP2A depletion significantly suppressed the anchorage-independent growth of both CNE-2 and SUNE-1 cells (all *P* < 0.05, Figure 3E). To further explore whether CIP2A was required for NPC tumor growth in vivo, we conducted xenograft tumor model assays by subcutaneously injecting SUNE-1 cells stably expressing shCIP2A or scrambled control siRNA into the dorsal flank of several mice. CIP2A depletion resulted in a significant reduction in tumor growth (*P* < 0.01, Figure 4A-B). The average tumor weight was also significantly decreased in the CIP2A depletion group compared to the scrambled control siRNA-treated group (0.31 ± 0.11 g vs. 0.54 ± 0.24 g; *P* < 0.01, Figure 4C-D).

## Discussion

In this study, CIP2A was upregulated in both NPC cell lines and clinical samples, and those NPC patients with high CIP2A expression exhibited the poorest survival rates. Furthermore, silencing CIP2A expression influenced MYC protein expression and further suppressed NPC cell proliferation and tumor growth. Our results demonstrate that the overexpression of CIP2A plays important roles in the development and progression of NPC.

Reversible protein phosphorylation is one of the most important biological mechanisms for signal transduction, which is tightly regulated by protein kinases and phosphatases to maintain the balance of the protein’s phosphorylation status and control its biological functions. However, there is considerable evidence indicating that the perturbation of this balance, including the activation of protein kinases and inhibition of phosphatases, contributes to the origin and pathogenesis of several human diseases, including cancer [21]. Protein phosphatase 2A (PP2A) is one important type of serine/threonine phosphatase; PP2A is inhibited in human cancers and functions as a tumor suppressor. Furthermore, the inhibition of PP2A activity has been found to result in the immortalization and malignant transformation of human cells [22,23]. Interestingly, CIP2A has recently been identified as an endogenous PP2A inhibitor in human cancer cells using the tandem affinity purification method. In addition, CIP2A inhibition has been found to enhance the catalytic phosphatase activity of the PP2A complex in several types of human malignancies [6,24]. Furthermore, CIP2A also exhibits the ability to transform human immortalized cells [6]; these results expand the general understanding of the mechanisms that are critical for cancer development and progression.

CIP2A was previously demonstrated to be a human oncoprotein due to its ability to transform human immortalized cells. Recently, CIP2A was found to be overexpressed at high frequencies in several types of human cancers [6-11]. More importantly, several studies reported that CIP2A could serve as a prognostic indicator for various solid and hematological tumors, including non-small cell lung cancer [12], colon cancer [13], breast cancer [14], ovarian cancer [15], renal cancer [16], tongue cancer [17], esophageal adenocarcinoma [18], bladder cancer [19], and chronic myeloid leukemia [20]. In the present study, CIP2A was significantly overexpressed in NPC cell lines and clinical specimens at both the mRNA and protein levels. Strikingly, NPC patients with high CIP2A protein expression had poorer overall and disease-free survival rates than those with low CIP2A protein expression. Multivariate Cox regression analysis demonstrated that low CIP2A protein expression was an independent prognostic indicator in patients with NPC. These results suggest that CIP2A expression status can serve as a valuable prognostic biomarker to stratify NPC patients into different risk groups and further guide individual therapy choices for patients with NPC.

In addition to its biological significance in the promotion of malignant transformation of human cells, CIP2A also plays important roles in carcinogenesis and the progression of human cancers. Several recent studies have reported that silencing CIP2A decreases cell viability and suppresses anchorage-independent growth in several types of human cancer cells [6-11]. It also promotes progenitor cell self-renewal and protects cancer cells from therapy-induced apoptosis or the induction of senescence [25,26]. A recent study demonstrated that CIP2A can regulate the cell cycle by targeting PLK1 [27]. More importantly, recent studies have also demonstrated that the depletion of CIP2A via siRNAs inhibits xenograft tumor growth [6,9]. In our present study, we also depleted CIP2A expression via siRNA to better understand the function of CIP2A in NPC. Inhibition of CIP2A expression significantly inhibited NPC cell viability and proliferation in vitro. Furthermore, silencing CIP2A suppressed xenograft tumor growth in vivo. Taken together, these results demonstrate that the dysregulation of CIP2A may contribute to the development and progression of NPC.

In addition, the depletion of CIP2A expression via siRNA suppressed MYC protein expression in NPC cell lines. MYC is one of the most-studied oncogenes, and it is involved in several malignant cellular processes. CIP2A can inhibit the degradation of MYC and therefore enhance its oncogenic activities by inhibiting the PP2A-mediated dephosphorylation of MYC at serine 62 [6-8]. CIP2A and MYC are regulated by a positive feedback loop that promotes the expression of both proteins [7]. Furthermore, the mechanisms of CIP2A activation and overexpression in cancer cells has been investigated by several other studies in which E2F1, ETS1, and ATF2 were found to directly bind to the CIP2A promoter and further stimulate CIP2A transcription [28-30]. Based on the functions and mechanisms of CIP2A activation in human cancers, the therapeutic targeting of CIP2A could facilitate a novel strategy for cancer therapy, including the use of CIP2A small RNA interference technology or the development of small molecules that target the CIP2A-PP2A interaction [6,31]. In addition, another alternative strategy to inhibit CIP2A activity is to target the signaling mechanisms that drive high CIP2A expression, such as the use of MYC [7], EGFR [29], and MEK inhibitors [29].

## Conclusions

In conclusion, the present study indicated that CIP2A overexpression was associated with poor survival in patients with NPC, and the depletion of CIP2A expression could inhibit cell viability and growth by promoting the stability of the CIP2A protein. Our findings provide new insights into the molecular mechanisms involved in the regulation of NPC progression and provide novel therapeutic targets and strategies for the treatment of NPC patients.

## Materials and methods

### Cell culture

Human NPC cell lines (CNE-1, CNE-2, SUNE-1, C666-1, HNE1, and HONE1) were grown in RPMI-1640 (Invitrogen) medium supplemented with 10% fetal bovine serum (Gibco). The immortalized nasopharyngeal epithelial cell line NP69 was cultured in keratinocyte/serum-free medium (Invitrogen) supplemented with bovine pituitary extract. The 293FT cell line was maintained in DMEM (Invitrogen) supplemented with 10% fetal bovine serum.

### Clinical specimens

Eighteen freshly frozen NPC specimens and fourteen normal nasopharyngeal epithelium samples were obtained from Sun Yat-sen University Cancer Center. In addition, we collected 280 paraffin-embedded NPC specimens from our hospital between January 2003 and February 2006. None of the patients received any anti-tumor therapy prior to the biopsy sample collection. The clinical features of all patients are provided in Table 1. TNM staging was performed according to the 7^th^ Edition of the AJCC/UICC Cancer Staging Manual. All patients were treated with conventional two-dimensional radiotherapy, and patients with stage III-IV disease also received platinum-based concurrent chemotherapy [32]. The median follow-up time was 63.6 months (range: 5.2-91.87). This study was approved by the Institutional Ethical Review Board of Sun Yat-sen University Cancer Center, and written informed consent was obtained from each patient.

### RNA extraction, reverse transcription, and quantitative PCR

Total RNA was isolated using TRIzol reagent (Invitrogen) and reverse-transcribed using M-MLV reverse transcriptase (Promega) and random primers (Promega). Quantitative PCR reactions using a Platinum SYBR Green qPCR SuperMix-UDG reagent (Invitrogen) were performed with a Bio-Rad CFX96 sequence detection system (Bio-Rad Laboratories Inc.). The following primers were used for CIP2A: 5′-CCATATGCTCACTCAGATGATGT-3′ (forward) and 5′-GTGTATCATCTCCACAGAGAGTT-3′ (reverse). Primers for GAPDH were as follows: 5′-CTCCTCCTGTTCGACAGTCAGC-3′ (forward) and 5′-CCCAATACGACCAAATCCGTT -3′ (reverse). Reactions containing either no template or no reverse transcriptase were used as negative controls. GAPDH was used as the normalization control, and the relative expression levels were calculated by the 2^-ΔΔCT^ method [33].

### Western blot analysis

Total protein was extracted with sample buffer (62.5 mmol/L Tris–HCl, pH 6.8, 2% SDS, 10% glycerol, and 5% 2-β-mercaptoethanol), and its concentration was quantified using the Pierce® BCA Protein Assay Kit (Thermo). Total protein was subsequently separated on 10% SDS-PAGE gels and transferred onto polyvinylidene fluoride membranes (Millipore). The membranes were blocked with 5% skim milk and incubated with primary antibodies recognizing CIP2A (1:1,000; Abcam) and MYC (1:5,000; Epitomics), followed by incubation with anti-mouse or rabbit IgG secondary antibodies (1:5,000; Sigma). Bands were detected by enhanced chemiluminescence, and GAPDH levels (1:5,000; Epitomics antibody) served as the loading control.

### Immunohistochemistry

Sections obtained from 280 paraffin-embedded NPC specimens were tested for CIP2A expression by immunohistochemical staining, as previously described [34,35]. Briefly, samples were deparaffinized and rehydrated, and the endogenous peroxidase activity was quenched. Antigen retrieval was performed, and the sections were blocked with bovine serum albumin and subsequently incubated with an anti-CIP2A antibody (1:200; Abcam). Sections were washed and subsequently incubated with a biotinylated secondary antibody bound to a streptavidin-horseradish peroxidase complex and visualized with 3,3-diaminobenzidine. All sections were scored by two independent pathologists, and the staining index was calculated as the product of the staining intensity (1, no staining; 2, weak staining; 3, moderate staining; 4, strong staining) and the proportion of positive cells (1, <10%; 2, 10-35%; 3, 35-70%; 4, >70%).

### Small interfering RNA transfection and the generation of stably transfected cell lines

Double-stranded small interfering RNA targeting CIP2A (50 nM, GenePharma; siCIP2A 1 5′-CUGUGGUUGUGUUUGCACUTT-3′; siCIP2A 2 5′-ACCAUUGAUAUCCUUAGAATT -3′) or scrambled control siRNA (5′-UAACAAUGAGAGCACGGCTT-3′) were transfected into CNE2 and SUNE1 cell lines using Lipofectamine 2000 reagent (Invitrogen). The CIP2A short hairpin RNA (shRNA) was synthesized and cloned into a pSUPERretro-puromycin plasmid using Bgl II and EcoR I enzymes (New England Biolabs). The pSUPERretro-shCIP2A plasmid or empty vector was co-transfected into 293FT cells along with the retroviral packaging vector PIK. After transfection, the supernatants were harvested and used to infect SUNE1 cells, and the stably transfected cells were selected with puromycin and validated by western blot analysis.

### Immunofluorescence staining

CNE-2 and SUNE-1 cells were grown on coverslips (Fisher Scientific). After 24 h, cells were incubated with primary antibodies against CIP2A (1:200; Abcam) and MYC (1:200; Epitomics), and subsequently incubated with Alexa Fluor® 488 or 594 goat anti-mouse or anti-rabbit IgG antibodies (Life Technologies). The coverslips were counterstained with DAPI, and the images were captured using a confocal laser-scanning microscope (Olympus FV1000).

### MTT assay

CNE-2 and SUNE-1 cells were seeded in 96-well plates at a density of 1,000 cells per well. At 1, 2, 3, 4, and 5 days, the cells were stained with 20 μl of MTT dye (0.5 mg/ml, Sigma) for 4 h, after which the medium was removed, and 100 μl of dimethyl sulfoxide (Sigma) was added. The absorbance was measured at 490 nm with a spectrophotometric plate reader.

### Colony formation assay

CNE-2 and SUNE1 cells were seeded in six-well plates at a density of 500 cells per well and cultured for 7 or 12 days. Colonies were fixed with 4% paraformaldehyde solution, stained with 0.5% crystal violet, and counted under an inverted microscope.

### Anchorage-independent soft-agar growth

CNE-2 and SUNE-1 cells (2.5 × 10^4^) were suspended in 1 ml of complete medium containing 0.66% agar (Sigma) and then applied to the top of a 1% agar/complete medium layer in six-well plates. Colonies were counted under an inverted microscope after 9 or 12 days.

### Xenograft tumor model

Three- to four-week-old male BALB/c nude mice were purchased from the Medical Experimental Animal Center of Guangdong Province (Guangzhou, China). All experimental animal protocols were approved by the Animal Care and Use Ethics Committee. SUNE-1 cells stably expressing shCIP2A or scrambled control shRNA were suspended in PBS, and 1 × 10^6^ cells (100 μl) were subcutaneously injected into the dorsal flank of each mouse. Tumors were examined every 3 days, and tumor volumes were calculated. On day 28, the mice were sacrificed, and the tumors were dissected and weighted.

### Statistical analysis

Data are presented as the mean ± SD, and differences between groups were analyzed using Student’s *t*-test or a chi-squared test. Receiver operation characteristic (ROC) curves were used to determine the optimal cutoff values for low and high CIP2A expression. The Kaplan-Meier method and log-rank test were used to estimate survival rates, and hazard ratios (HRs) were calculated using unadjusted univariate Cox regression analysis. Multivariate Cox regression analysis was used to test for independent prognostic factors. All statistical analyses were performed with SPSS 16.0 software, and *P* values of < 0.05 were considered statistically significant.

## Competing interests

The authors declare that they have no competing interests.

## Authors’ contributions

NL, QH, JC, YL, YX, and XR performed experiments; JM, NL, QH, JC, QL YX, XR, YS, HM, JS, WJ, TK, and MZ designed research, analyzed data and edited the manuscript for intellectual content. All authors have made critical edits to the manuscript and have given final approval.

## References

[B1] Xu ZJ Zheng RS Zhang SW Zou XN Chen WQ Nasopharyngeal carcinoma incidence and mortality in China in 2009 Chin J Cancer 2013 32 453 460 10.5732/cjc.013.10118 23863562 PMC3845582

[B2] Cho WC Most common cancers in Asia-Pacific region: nasopharyngeal carcinoma Cancer report of Asian-Pacific region 2010 2010 Asian Pacific Organization for Cancer Prevention 284 289

[B3] Jemal A Bray F Center MM Ferlay J Ward E Forman D Global cancer statistics CA Cancer J Clin 2011 61 69 90 10.3322/caac.20107 21296855

[B4] Lai SZ Li WF Chen L Luo W Chen YY Liu LZ Sun Y Lin AH Liu MZ Ma J How does intensity-modulated radiotherapy versus conventional two-dimensional radiotherapy influence the treatment results in nasopharyngeal carcinoma patients? Int J Radiat Oncol Biol Phys 2011 80 661 668 10.1016/j.ijrobp.2010.03.024 20643517

[B5] Chen Y Sun Y Liang SB Zong JF Li WF Chen M Chen L Mao YP Tang LL Guo Y Lin AH Liu MZ Ma J Progress report of a randomized trial comparing long-term survival and late toxicity of concurrent chemoradiotherapy with adjuvant chemotherapy versus radiotherapy alone in patients with stage III to IVB nasopharyngeal carcinoma from endemic regions of China Cancer 2013 119 2230 2238 10.1002/cncr.28049 23576020

[B6] Junttila MR Puustinen P Niemelä M Ahola R Arnold H Böttzauw T Ala-aho R Nielsen C Ivaska J Taya Y Lu SL Lin S Chan EK Wang XJ Grènman R Kast J Kallunki T Sears R Kähäri VM Westermarck J CIP2A inhibits PP2A in human malignancies Cell 2007 130 51 62 10.1016/j.cell.2007.04.044 17632056

[B7] Khanna A Böckelman C Hemmes A Junttila MR Wiksten JP Lundin M Junnila S Murphy DJ Evan GI Haglund C Westermarck J Ristimäki A MYC-dependent regulation and prognostic role of CIP2A in gastric cancer J Natl Cancer Inst 2009 101 793 805 10.1093/jnci/djp103 19470954

[B8] Niemelä M Kauko O Sihto H Mpindi JP Nicorici D Pernilä P Kallioniemi OP Joensuu H Hautaniemi S Westermarck J CIP2A signature reveals the MYC dependency of CIP2A-regulated phenotypes and its clinical association with breast cancer subtypes Oncogene 2012 31 4266 4278 10.1038/onc.2011.599 22249265

[B9] Côme C Laine A Chanrion M Edgren H Mattila E Liu X Jonkers J Ivaska J Isola J Darbon JM Kallioniemi O Thézenas S Westermarck J CIP2A is associated with human breast cancer aggressivity Clin Cancer Res 2008 15 5092 5100 19671842 10.1158/1078-0432.CCR-08-3283

[B10] Li W Ge Z Liu C Liu Z Björkholm M Jia J Xu D CIP2A is overexpressed in gastric cancer and its depletion leads to impaired clonogenicity, senescence, or differentiation of tumor cells Clin Cancer Res 2008 14 3722 3728 10.1158/1078-0432.CCR-07-4137 18559589

[B11] Puustinen P Rytter A Mortensen M Kohonen P Moreira JM Jäättelä M CIP2A oncoprotein controls cell growth and autophagy through TORC1 activation J Cell Biol 2014 204 713 727 10.1083/jcb.201304012 24590173 PMC3941044

[B12] Dong QZ Wang Y Dong XJ Li ZX Tang ZP Cui QZ Wang EH CIP2A is overexpressed in non-small cell lung cancer and correlates with poor prognosis Ann Surg Oncol 2011 18 857 865 10.1245/s10434-010-1313-8 20842459

[B13] Wiegering A Pfann C Uthe FW Otto C Rycak L Mäder U Gasser M Waaga-Gasser AM Eilers M Germer CT CIP2A influences survival in colon cancer and is critical in maintaining Myc expression PLoS One 2013 8 e75292 10.1371/journal.pone.0075292 24098375 PMC3788051

[B14] Yu G Liu G Dong J Jin Y Clinical implications of CIP2A protein expression in breast cancer Med Oncol 2013 30 524 23471718 10.1007/s12032-013-0524-9

[B15] Böckelman C Lassus H Hemmes A Leminen A Westermarck J Haglund C Bützow R Ristimäki A Prognostic role of CIP2A expression in serous ovarian cancer Br J Cancer 2011 105 989 995 10.1038/bjc.2011.346 21897396 PMC3185957

[B16] Ren J Li W Yan L Jiao W Tian S Li D Tang Y Gu G Liu H Xu Z Expression of CIP2A in renal cell carcinomas correlates with tumour invasion, metastasis and patients’ survival Br J Cancer 2011 105 1905 1911 10.1038/bjc.2011.492 22075943 PMC3251889

[B17] Böckelman C Hagström J Mäkinen LK Keski-Säntti H Häyry V Lundin J Atula T Ristimäki A Haglund C High CIP2A immunoreactivity is an independent prognostic indicator in early-stage tongue cancer Br J Cancer 2011 104 1890 1895 10.1038/bjc.2011.167 21610708 PMC3111200

[B18] Rantanen T Kauttu T Akerla J Honkanen T Krogerus L Salo J Paavonen T Oksala N CIP2A expression and prognostic role in patients with esophageal adenocarcinoma Med Oncol 2013 30 684 23925667 10.1007/s12032-013-0684-7

[B19] Xue Y Wu G Wang X Zou X Zhang G Xiao R Yuan Y Long D Yang J Wu Y Xu H Liu F Liu M CIP2A is a predictor of survival and a novel therapeutic target in bladder urothelial cell carcinoma Med Oncol 2013 30 406 23275123 10.1007/s12032-012-0406-6

[B20] Lucas CM Harris RJ Giannoudis A Copland M Slupsky JR Clark RE Cancerous inhibitor of PP2A (CIP2A) at diagnosis of chronic myeloid leukemia is a critical determinant of disease progression Blood 2011 117 6660 6668 10.1182/blood-2010-08-304477 21490338

[B21] Khanna A Pimanda JE Westermarck J Cancerous inhibitor of protein phosphatase 2A, an emerging human oncoprotein and a potential cancer therapy target Cancer Res 2013 73 6548 6553 10.1158/0008-5472.CAN-13-1994 24204027

[B22] Jansens V Goris J Hoof C PP2A: the expected tumor suppressor Curr Opin Genet Dev 2005 15 34 41 10.1016/j.gde.2004.12.004 15661531

[B23] Rangarajan A Hong SJ Gifford A Weinberg RA Species- and cell type-specific requirements for cellular transformation Cancer Cell 2004 6 171 183 10.1016/j.ccr.2004.07.009 15324700

[B24] Chen KF Liu CY Lin YC Yu HC Liu TH Hou DR Chen PJ Cheng AL CIP2A mediates effects of bortezomib on phospho-Akt and apoptosis in hepatocellular carcinoma cells Oncogene 2010 29 6257 6266 10.1038/onc.2010.357 20729919

[B25] Kerosuo L Fox H Perälä N Ahlqvist K Suomalainen A Westermarck J Sariola H Wartiovaara K CIP2A increases self-renewal and is linked to Myc in neural progenitor cells Differentiation 2010 80 68 77 10.1016/j.diff.2010.04.003 20447748

[B26] Tseng LM Liu CY Chang KC Chu PY Shiau CW Chen KF CIP2A is a target of bortezomib in human triple negative breast cancer cells Breast Cancer Res 2012 14 R68 10.1186/bcr3175 22537901 PMC3446403

[B27] Kim JS Kim EJ Oh JS Park IC Hwang SG CIP2A modulates cell cycle progression in human cancer cells by regulating the stability and activity of PLK1 Cancer Res 2013 73 6667 6678 10.1158/0008-5472.CAN-13-0888 23983103

[B28] Laine A Sihto H Come C Rosenfeldt MT Zwolinska A Niemelä M Khanna A Chan EK Kähäri VM Kellokumpu-Lehtinen PL Sansom OJ Evan GI Junttila MR Ryan KM Marine JC Joensuu H Westermarck J Senescence sensitivity of breast cancer cells is defined by positive feedback loop between CIP2A and E2F1 Cancer Discov 2013 3 182 197 10.1158/2159-8290.CD-12-0292 23306062 PMC3572190

[B29] Khanna A Okkeri J Bilgen T Tiirikka T Vihinen M Visakirpi T Westermarck J ETS2 mediates MEK1/2-dependent overexpression of cancerous inhibitor of protein phosphatase 2A (CIP2A) in human cancer cells PLoS One 2011 6 e17979 10.1371/journal.pone.0017979 21445343 PMC3062549

[B30] Mathiasen DP Egebjerg C Andersen SH Rafn B Puustinen P Khanna A Daugaard M Valo E Tuomela S Bottzauw T Nielsen CF Willumsen BM Hautaniemi S Lahesmaa R Westermarck J Jäättelä M Kallunki T Identification of a c-Jun N-terminal kinase-2-dependent signal amplification cascade that regulates c-Myc levels in ras transformation Oncogene 2012 31 390 401 10.1038/onc.2011.230 21706057

[B31] Pommier Y Marchand C Interfacial inhibitors: targeting macromolicular complexes Nat Rev Drug Discov 2012 11 25 36 22173432 10.1038/nrd3404 PMC7380715

[B32] Chan AT Teo PM Ngan PK Leung TW Lau WH Zee B Leung SF Cheung FY Yeo W Yiu HH Yu KH Chiu KW Chan DT Mok T Yuen KT Mo F Lai M Kwan WH Choi P Johnson PJ Concurrent chemotherapy-dadiotherapy compared with radiotherapy alone in locoregionally advanced nasopharyngeal carcinoma: progression-free survival analysis of a phase III randomized trial J Clin Oncol 2002 20 2038 2044 10.1200/JCO.2002.08.149 11956263

[B33] Livak KJ Schmittgen TD Analysis of relative gene expression data using real-time quantitative PCR and the 2(-Delta Delta C(T)) Method Methods 2001 25 402 408 10.1006/meth.2001.1262 11846609

[B34] Liu N Cui RX He QM Huang BJ Sun Y Xie D Zeng J Wang HY Ma J Reduced expression of Dicer1 is associated with poor prognosis in patients with nasopharyngeal carcinoma Med Oncol 2013 30 360 23307239 10.1007/s12032-012-0360-3

[B35] Liu N Tang LL Sun Y Cui RX Wang HY Huang BJ He QM Jiang W Ma J MiR-29c suppresses invasion and metastasis by targeting TIAM1 in nasopharyngeal carcinoma Cancer Lett 2013 329 181 188 10.1016/j.canlet.2012.10.032 23142282

